# Supplementary material for: Patterns of Intron Gain and Loss in Fungi
Source: PLoS Biol. 2004 Nov 30;2(12):e422. doi: 10.1371/journal.pbio.0020422 (PMC532390; doi:10.1371/journal.pbio.0020422)
Supplement: Table S1 — Also available at http://genes.mit.edu/NielsenEtAl/. (4.3 MB ZIP). [file pbio.0020422.st001.zip › NielsenEtAl/html/127.html]

AN1194.1.NCU09896.1.MG06348.1.FG01329.1


```
 CLUSTAL W (1.82) Multiple Sequence Alignments - Introns Inserted


Sequence 1: MG06348.1	208 aa
Sequence 2: FG01329.1	207 aa
Sequence 3: NCU09896.1	211 aa
Sequence 4: AN1194.1	206 aa
Alignment Length: 211 aa
Number Identitical Residues: 122 aa
Alignment Score (without introns) 5699


MG06348.1 	MAT2NITWHDGLTRRERNQLRGQRGFTIWFTGLSASGKSTVATALEQHLLHLGLAAYRLD
NCU09896.1	MAT2NITWHPSLSRHERNQLRGQRGFTIWFTGLSASGKSTVATALEQHLLHIGLAAYRLD
FG01329.1 	MAT2NITWHPSLSRKEREETRGQRGLTIWLTGLSASGKSTVATALEQHLLHLGVAAYRLD
AN1194.1  	MAT2NITHHAGITRNERNQLRKQKGLTIWLTGLSASGKSTIAVELEHQLLQRGLHAYRLD
          	*** *** * .::*.**:: * *:*:***:**********:*. **::**: *: *****

MG06348.1 	GDNVRFGLNKDLGFSEKDRNENIRRIAE~VAKLFADSSVVALTSFISPYRADRQTARELH
NCU09896.1	GDNVRFGLNKDLGFSEKDRNENIRRIAE0VAKLFADSATIALTSFISPYRADRQIARDLH
FG01329.1 	GDNVRFGLNKDLGFSEVDRNENIRRISE~VAKLFADSSTIAITSFISPYRADRQVARQLH
AN1194.1  	GDNVRFGLNKDLGFSDADRNENIRRIAE~VAKLFADSSSIAITSFISPFRADRDTARKLH
          	***************: *********:* ********: :*:******:****: **.**

MG06348.1 	AQAAQKGDEPLEFVEVYVDIPIEVAEQRDPKGLYKKARAGEIKEFTGISAPYEAPENPEI
NCU09896.1	ANATQAGDDVLPFIEVFVDVPLAEAEKRDPKGLYKKARAGEIKDFTGISAPYEAPESPEI
FG01329.1 	EQATQGGDAPIPFVEVYVDVPLEVAEQRDPKGLYKKARAGEIKDFTGISAPYEEPEKAEI
AN1194.1  	EVPTPNDSTGLPFVEVFVDVPIEVAEKRDPKGLYKKAREGIIKEFTGISSPYEAPENPEV
          	  .:  ..  : *:**:**:*:  **:*********** * **:*****:*** **..*:

MG06348.1 	TIKTHENSVEECVAQIVKWLQDKGLVKATP---
NCU09896.1	TIRTDLLSVEECVKKIVDYLAEAGLISGTKETR
FG01329.1 	TIKTHENSVEECVAQIVEWLNEKGYLNKK----
AN1194.1  	HVKNVDLPIQEAVKQIIDYLDSKKLLDA-----
          	 ::.   .::*.* :*:.:* .   :.
```
